# The Impact of Smoking on Microbiota: A Narrative Review

**DOI:** 10.3390/biomedicines11041144

**Published:** 2023-04-10

**Authors:** Sara Cicchinelli, Federico Rosa, Federica Manca, Christian Zanza, Veronica Ojetti, Marcello Covino, Marcello Candelli, Antonio Gasbarrini, Francesco Franceschi, Andrea Piccioni

**Affiliations:** 1 Department of Emergency, Ospedale SS. Filippo e Nicola, 67051 Avezzano, Italy; 2 Department of Emergency Medicine, Fondazione Policlinico Universitario, Università Cattolica del Sacro Cuore, 00168 Roma, Italy; 3 Department of Anesthesia, Critical Care, and Emergency Medicine, Ospedale Michele e Pietro Ferrero, 12060 Cuneo, Italy; 4 Department of Internal Medicine, Ospedale San Carlo di Nancy, 00165 Rome, Italy; 5 Department of Internal Medicine, Division of Gastroenterology, Fondazione Policlinico Universitario A. Gemelli, Università Cattolica del Sacro Cuore, 00168 Rome, Italy

**Keywords:** smoking, microbiota, cigarette smoke

## Abstract

Cigarette smoke is a classic risk factor for many diseases. The microbiota has been recently indicated as a new, major player in human health. Its deregulation—dysbiosis—is considered a new risk factor for several illnesses. Some studies highlight a cross-interaction between these two risk factors—smoke and dysbiosis—that may explain the pathogenesis of some diseases. We searched the keywords “smoking OR smoke AND microbiota” in the title of articles on PubMed^®^, UptoDate^®^, and Cochrane^®^. We included articles published in English over the last 25 years. We collected approximately 70 articles, grouped into four topics: oral cavity, airways, gut, and other organs. Smoke may impair microbiota homeostasis through the same harmful mechanisms exerted on the host cells. Surprisingly, dysbiosis and its consequences affect not only those organs that are in direct contact with the smoke, such as the oral cavity or the airways, but also involve distant organs, such as the gut, heart, vessels, and genitourinary tract. These observations yield a deeper insight into the mechanisms implicated in the pathogenesis of smoke-related diseases, suggesting a role of dysbiosis. We speculate that modulation of the microbiota may help prevent and treat some of these illnesses.

## 1. Introduction

“Do you smoke? How many cigarettes do you smoke daily? Since when?”, we often ask these questions to our patients. Physicians have been taught that cigarette smoke is a risk factor for many diseases. It affects the pathogenesis of cardiovascular and respiratory diseases, oncogenesis, autoimmune, and immune-mediated illnesses [1]. In the past years, many mechanisms have been identified as responsible for the noxious effects of cigarette smoke. On one hand, toxic substances released by combustion have a direct effect on genetic mutations; on the other, they promote a pro-inflammatory environment that damages distant organs as well [2].

Despite the fact that this evidence has been known for a long time, the number of smokers worldwide remains extremely high, thus, leading us to think that this issue will not be solved soon [3]. Recently, besides “classic” risk factors such as smoke, a new, interesting player in human health and disease is gaining the attention of researchers: the microbiota [4]. The term microbiota describes the microbial populations living in the human body. It includes not only bacteria, but also archaea and viruses. The microbiota of the gut is the most abundant, consisting of over one thousand resident microorganisms, mostly bacteria. Among these bacteria, the main phyla are *Firmicutes*, *Proteobacteria*, and *Bacteroidetes* [5]. The relationship between the microbiota and its host is as complex as it is fascinating: it is established from the earliest moments of life, then influenced over the years by multiple environmental factors, such as diet, physical activity, genetics, chemical, and physical agents [4]. In health conditions, most of the bacteria of the microbiota are commensals: this condition is referred to as “eubiosis”. This means that, while obtaining their habitat and nourishment from the host, these microbes protect the host from other pathogens, preventing infections. In fact, their interaction with the epithelial surface works as a physical barrier, increasing the competition for nutrients, producing antimicrobial peptides, and modulating immune cell function both in a pro- and anti-inflammatory fashion [5,6]. The imbalance in the composition of the normal microbiota, under the effect of environmental factors, is defined as “dysbiosis”. Dysbiosis has an important impact on the immune system function and, consequently, affects the susceptibility and the clinical course of many diseases [4,5,7]. In the first place, the study of the role of the microbiota has involved those organs that were obviously not sterile, first of all, the gut. This was also related to the detection techniques, relying on cultural methods. Lately, the growing interest of researchers in the microbiota has spread because of technological advances—such as new gene sequencing techniques—that have led to the identification of resident bacterial populations in almost all organs and systems of the body, even in those that were once considered sterile [8]. While clarifying the role of the microbiota in health and disease, scientists have starting questioning the possibility to intervene by correcting this dysbiosis with the many weapons we have at our disposal, such as probiotics and prebiotics, right up to the more invasive faecal microbiota transplantation [6].

It is curious to note how some diseases that have been classically related to tobacco smoke exposure have been recently associated with dysbiosis.

Among these illnesses, we can enumerate cardiovascular diseases such as myocardial infarction and myopericarditis, respiratory diseases such as asthma and COPD (chronic obstructive pulmonary disease), neurological diseases such as stroke, Alzheimer’s, and Parkinson’s disease, gastrointestinal diseases such as IBDs (inflammatory bowel diseases), autoimmune illnesses such as rheumatoid arthritis, and also mental issues such as depression, dementia, and autism [4,5,6].

Therefore, the aim of this review of the literature is to understand the relationship between these two topics—smoking and microbiota—since this may allow us to comprehend the pathogenesis of several diseases. Moreover, in the future, this knowledge may be a starting point to prevent and treat them.

## 2. Materials and Methods

This narrative review includes studies published in English over the last 25 years, on the topics of microbiota and cigarette smoking, focusing on the respiratory and gastrointestinal tracts. We searched on PubMed^®^, UptoDate^®^, and Cochrane^®^. The keywords we searched for were “smoking OR smoke AND microbiota”, in the titles of articles. The inclusion and exclusion criteria are reported in Table 1. After the application of the inclusion and exclusion criteria we were able to select 51 articles. All the articles have been read, and their bibliography has been checked in order to select other works reputed as relevant based on the opinion of the authors. No ethical approval was required to perform this review.

## 3. Results

We selected 51 articles from the preliminary search. After the reading and reference revision of these articles, we have selected other valuable manuscripts, for a total of 64 papers. We have divided the articles into four sections: oral cavity, airways, gut, and other organs (Table 2).

### 3.1. Section I—Oral Cavity

The human oral cavity is everything but sterile. It hosts the second most abundant microbiota community of the human body, after the gut [3]. Bacteria, together with viruses and fungi, occupy different ecological niches, such as the teeth, gingival sulcus, tongue, cheeks, hard and soft palates, and tonsils (Table 3).

In 2010, Dewhirst et al. sequenced the human oral microbiome and included the 16S rRNA gene sequences into a curated, phylogeny-based web accessible database, named the Human Oral Microbiome Database (HOMD) [9]. The extended version of the database (eHOMD) reports that there are 774 oral bacterial species: 58% are officially named, 16% unnamed but cultivated, and 26% are known only as uncultivated phylotypes. The eHOMD taxonomy provides a provisional naming scheme for the currently unnamed taxa [10].

The identified bacteria belong to 13 phyla: Actinobacteria, Bacteroidetes, Chlamydiae, Chloroflexiare, Euryarchaeota, Firmicutes, Fusobacteria, Proteobacteria, Spirochaetes, SR1, Synergistetes, Tenericutes, and TM7. Approximately 96% of the taxa belongs to six major phyla: Firmicutes, Bacteroidetes, Proteobacteria, Actinobacteria, Spirochaetes, and Fusobacteria [9,10].

Oral dysbiosis may be promoted by many factors, such as host diet and nutrition, genetic predisposition, hormonal factors, antibiotic exposure, alcohol consumption, infections, and tobacco smoke.

It is known that oral dysbiosis is related not only to oral health issues, such as dental caries, periodontal diseases, and tooth loss, but also to systemic diseases, such as cardiovascular disease, diabetes, and even cancer [2].

Regarding the role of the microbiota in oral health, periodontal diseases are among the most studied issues.

Periodontal diseases are multifactorial diseases that arise from the interaction among the subgingival microbiota and environmental risk factors. Among the latter, smoking is a remarkable risk factor for periodontitis progression.

Classically, a triad of bacteria, called “the red complex”, is considered responsible for the pathogenesis of periodontitis: *Porphyromonas gingivalis*, *Tannerella forsythia*, and *Treponema denticola.* In addition to this triad, the “orange complex”, formed by *Fusobacterium*, *Prevotella*, and *Campylobacter* species, is also often associated with periodontitis [11,12]. In light of this, beside works describing the normal oral microbiota composition of healthy, non-smoker individuals [9,10], many authors have reported that, in healthy subjects, smoking affects the composition of the microbiota collected from various sites of the oral cavity, either via depletion of beneficial bacteria or via increase in pathogenic bacteria.

Analyzing the effect of smoke on samples from eight oral sites (i.e., keratinized gingiva, hard palate, buccal mucosa, palatine tonsil, tongue dorsum, supragingival and subgingival plaques, and saliva), and from nasal swabs of both nares, Yu et al. found that alpha diversity is lower in smokers than in non-smokers in the buccal mucosa, but not in other sample sites [13].

Additionally, healthy smokers have shown a higher prevalence of pathogens and a depletion of commensals.

Bašić et al. reported an increase of *Actinomyces odontolyticus*, and a significantly lower prevalence of *Streptococcus sanguinis* in smokers compared to non-smokers. *A. ondontolyticus* is a pathogen, since it adheres to the tooth surface and coagulates with other bacteria, leading to caries and pulpitis. Together with *Veillonella parvula*, they are nitrite-producing bacteria that form the “purple complex” of periodontitis, which seems to serve as a bridge to the orange and red complexes [14]. *S. sanguinis* is one of the first colonizers of the tooth surface, and is considered a beneficial bacterium because of its ability to produce hydrogen peroxide, which inhibits the adherence and growth of pathogens [12,13,14]. Similarly, Jia et al. reported the higher abundance *Actinomyces* and *Veillonella* in healthy smokers [15].

Mason et al. reported that, compared to healthy non-smokers, healthy smokers had a pathogen-rich, commensal-poor, anaerobic microbiota that resembles the disease-related one. In particular, smokers had a higher abundance of pathogens, mainly belonging to *Fusobacteria*, *Megasphaera*, and *Acinetobacteria* spp. Beneficial bacteria, including *S. sanguinis*, were depleted [16].

Aligned data were obtained by Haffajee and Socransky, who analyzed the microbiota of healthy never, former, and current smokers. The current smokers group had a higher prevalence of members of the orange and red complexes, including *Fusobacterium nucleatum*, *Prevotella intermedia*, *Prevotella nigrescens*, *Porphyromonas gingivalis*, and *Tannerella denticola* [11].

The study of members of the red and orange complex is also one of the latest frontiers of study regarding the microbiome of the oral cavity and its possible link to other extra-oral pathologies [17].

Even with subtle differences in the species of bacteria identified, other authors have described a depletion of commensals, and an increase of pathogens—not only belonging to the classical orange and complex—in healthy smokers [18,19,20].

Curiously, beside its role in enhancing the emergence of pathogens, smoke has also been associated with higher levels of two probiotics: *Bifidobacterium* and *Lactobacillus* [21]. This last observation may explain why current smokers show lower BMI values, since these two probiotic taxa have been associated with a decreased risk of obesity [21,22,23].

When studying patients with periodontitis and different smoking habits, other alterations may be observed.

The microbial community of smoking-associated periodontitis is less diverse and distinct from that of non-smokers, with a greater prevalence of pathogens. This finding has been associated with higher disease severity, especially in smokers [12,16].

Similar results have also been observed when comparing non-smokers and smokers affected by other oral issues, such as recurrent aphthous stomatitis [24] and caries [19,25].

Differences among healthy people and periodontitis, with relation to smoke habit, have also been observed in the profile of molecules expressed in the periodontal tissue, including antimicrobial peptides (AMPs), which are involved in the defence of mucosal surfaces.

Bunaes et al. measured the levels of markers involved in inflammatory pathways and bone remodelling mechanisms: interleukins (IL-1b, -2, -1ra, -4, -5, -6, -7, -8, -9, -10, -12, -13, -15, and -17), interferon (IFN)-c, basic fibroblast growth factor, granulocyte colony stimulating factor (G-CSF), eotaxin, granulocyte monocyte CSF (GM-CSF), IFN-inducible protein-10 (IP-10), monocyte chemo-attractive protein-1 (MCP-1), macrophage inflammatory protein (MIP)-1a, MIP-1b, platelet-derived growth factor (PDGF), regulated upon activation normally T expressed and presumably secreted (RANTES), tumour necrosis factor-a (TNF-a), and vascular endothelial growth factor (VEGF). Lower levels of these markers were observed among smokers, and this suggests an immunosuppressant effect of smoking on the local inflammatory response and bone remodelling [26].

Grant et al. analyzed the expression of AMPs in periodontal health and disease in the presence or absence of smoking habit. Seven AMPs were overabundant in periodontal disease in smokers: adrenomedullin, eosinophil peroxidase, three different histones, myeloperoxidase, and neutrophil defensin 1 [27].

In contrast with the previous findings, other authors have reported that while the composition of the microbiota differs based on the oral sampling location, it is minimally affected by the subject’s smoking habits, or by the presence or absence of periodontal disease. A trend was noted, with periodontitis and smoking subjects having higher proportions of periodontal pathogens [28].

Finally, it is interesting to remark that, even if smoke alters the composition of the oral microbiota, the effect may be reversible. In fact, studies comparing never smokers and former smokers have shown a similar composition of the microbiota in the two groups [21]. There are also contrasting studies assessing that previous smoking habits and periodontal disease may affect the peri-implant microbiota composition, even after many years [29].

In addition to classic smoking habits such as cigarettes and pipes, in recent years, electronic nicotine delivery devices (ENDDs) are more and more used. Data regarding their effects on the microbiota are scarce. Some authors have reported no significant difference in the alpha diversity, beta diversity, or taxonomic relative abundances when comparing electronic cigarette smokers and non-smokers [30].

A more recent study has shown that the use of electronic smoking enhances the emergence of opportunistic transient streptococci, namely, *S. pneumoniae* and *S. pyogenes,* with significant levels of colonization [31].

### 3.2. Section II—Airways

Studies of the lung microbiota are at early stages (Table 4). In particular, the lower respiratory tract has been long considered sterile. This misbelief relied on cultural techniques, which were not able to detect the resident microbes and, above all, anaerobes. With the advent of molecular techniques, such as the 16S rDNA-encoding gene sequence-based culture-independent techniques, the airway microbiota started to be characterized [32]. The fact that airways are not sterile is of pivotal importance when considering that the gut microbiota—the widest of the human body, accounting for the largest number of bacteria—is in contact with an epithelial surface of 30 m^2^, while the lung microbiota potentially occupies 40–80 m^2^ [33].

The normal airway microbiota includes *Actinobacteria*, *Proteobacteria*, *Bacteroidetes*, and *Firmicutes* [34]. At the genus level, *Prevotella*, *Neisseria*, *Haemophilus*, *Veillonella*, and *Streptococcus* are described as the most abundant in healthy populations [35].

These bacteria are commensals, and they seem to impact host resistance to pathogen colonization [35].

Many of these bacteria seem to come from the oral cavity. In fact, it is postulated that during feeding and respiration, microorganisms colonizing the oral cavity may spread on the epithelial surfaces of contiguous organs, such as the pharynx, nose, Eustachian tube and middle ear, sinuses, oesophagus, and, finally, the trachea, bronchi, and lungs [9].

However, some authors also hypothesize that the isolation of those bacteria may be the result of contamination from the passage of the bronchoscope through the oral cavity to obtain lung specimens [32].

Moreover, recent studies have highlighted that specific bacteria, such as *Enterobacteriaceae* and *Haemophilus* spp., are significantly more abundant in the lungs than would be expected if they originated from the mouth, demonstrating that the lung microbiome does not derive entirely from the oral cavity in healthy individuals [36].

Many factors, such as host genetics, and habits such as smoking, alcohol consumption, and physical activity, may alter the airway microbiota composition [35].

Smoking—even when passive—seems to have the strongest effect on the overall microbial community composition, since it can simultaneously deplete members of the normal commensal airway flora and enrich potential pathogens, with various mechanisms which resembles the ones reported for the oral cavity [35,37,38]. First of all, cigarette smoke contains potential respiratory pathogens, including *Acinetobacter*, *Clostridium*, *Klebsiella*, *Pseudomonas aeruginosa*, and *Serratia* spp. Moreover, smoking enhances bacterial adhesion to respiratory epithelial cells, disrupts the mucociliary clearance, and impairs the host immune responses [35,37,38]. The pathogenetic effect of tobacco smoke on the respiratory tract is a consolidated axiom.

Lately, airway dysbiosis—regardless of smoking habit—has been associated with a variety of illnesses, including asthma [33,34,39,40], respiratory infections [39,40,41], COPD (chronic obstructive pulmonary disease) [40], and even lung cancer [42].

Moreover, airway disease may be influenced by gut dysbiosis, because of the existence of a “gut–lung axis” [40,43].

When studying the influx of smoking on the airway microbiota, researchers have used the same model of the oral cavity, comparing the microbiota of different tracts of the airways of healthy smokers and non-smokers, and the microbiota of ill patients with different smoking habits. The results are far from consistent, homogeneous, and concordant.

In the upper respiratory tract, smoke seems to cause important changes in the microbiota composition. Charlson et al. analyzed samples of the right and left nasopharynx and oropharynx of 29 smoking and 33 non-smoking healthy asymptomatic adults [38].

The nasopharynx of healthy non-smokers was characterized mainly by sequences belonging to the *Firmicutes* phyla (73%), with *Proteobacteria*, *Bacteriodetes*, and *Actinobacteria* accounting for almost all of the remaining sequences. In the nasopharynx of smokers, the authors reported an increase in *Haemophilus* spp., even if modest. Other increased species included the *Actinomyces* species of *Eggerthella*, and the *Firmicutes* species *Erysipelotrichaceae I.S.*, *Dorea*, *Anaerovorax*, *Eubacterium*, and *Abiotrophia* spp. All of these genera contain Gram-positive anaerobic lineages, also associated with oral infections and occasionally with endocarditis.

In the oropharynx of non-smokers, sequences related to *Bacteroidetes* were most abundant (36.4%), while *Firmicutes* were less represented (27.7%). *Proteobacteria* taxa were detected in similar proportions in both airway sites (∼12%). In the oropharynx of smokers, the greatest increase was in *Megasphaera* spp., an anaerobic Gram-negative lineage of the *Firmicutes* phyla, which is known to reside in the oral cavity and is associated with periodontitis. Other increased potential pathogens belonged to *Streptococcus*, *Veillonella*, and *Actinomyces* spp. In contrast, the *Peptostreptococcus* genus decreased in smokers, which is interesting because many species of this genus are known to inhibit the growth of pathogens [31]. Similar results have been reported in a large study of the Australian population [33].

With regard to the lower respiratory tract of healthy individuals, Morris et al. reported that smoking influences the composition of the oral microbiota, but there were no significant differences in the lung microbiota of healthy smokers and non-smokers [32]. *Bacteroidetes* and *Proteobacteria* had a decreased relative abundance in bronchoalveolar lavage (BAL) from smokers, suggesting that smoke can cause subtle changes in healthy subjects [32].

In general, we can state that in smokers, bacterial communities were significantly more diverse than those of non-smokers, with a greater abundance of both known pathogens and organisms not previously recognized as associated with disease in smokers. In particular, the distributions of several genera were systematically altered by smoking, and there was an increase of anaerobic lineages [33,35,38].

With regard to airway pathology, there is evidence, even if fragmentary, about the interaction between smoke and the microbiota in the onset and progression of several diseases.

It is known that chronic exposure to tobacco smoke is a notable risk factor for COPD development. In addition to the direct effects of smoke on airways, studies have revealed a different microbiota composition between non-smoker COPD subjects and patients who were smokers with COPD. The nasal microbiota of COPD smokers was characterized by an increase in the abundance of pathogens, such as *Actinomyces*, *Actinobacillus*, *Megasphaera*, and *Selenomonas* spp. [44]. Other authors report an increased prevalence of *Haemophilus* spp. in COPD, regardless of their smoking habit [45].

The microbiota plays an early role in the development of asthma. The “hygiene hypothesis” suggests that the loss of microbial exposure in childhood allows asthma onset because of two reasons. First, signals from commensal organisms that normally downregulate mucosal immune responses are reduced; second, potential pathobionts enhance inflammation with subsequent mucosal damage.

Once the pathology is established, one of the most interesting findings about the asthmatic microbiota concerns the increase in *Proteobacteria,* such as *Neisseria* and *Haemophilus* spp., and the decrease of *Bacteroidetes,* such as *Prevotella* spp. [33,46]. Moreover, the microbiota—in particular *Streptococcus pneumoniae*, *Haemophilus influenzae*, *Moraxella catarrhalis*, *Chlamydia pneumoniae*, and *Mycoplasma pneumoniae*—is involved in the recurrence, severity, and response to therapy of exacerbations [46].

Studies on mice suggest that previous exposure to smoke is a risk factor for more severe pneumococcal infections, and that the infection itself causes modifications of the lung microbiota [47,48].

An interesting study on trauma patients developing an acute respiratory distress syndrome (ARDS) in the ICU has highlighted that potential pathogens, including *Streptococcus*, *Fusobacterium*, *Prevotella*, *Haemophilus*, and *Treponema*, are more likely to be found in smokers. ARDS development was associated with the lung community composition at baseline and at 48 h [49].

In light of the above, we agree with the other authors in emphasising the fundamental role played by the “gut-lung axis”. [40,43,50,51].

### 3.3. Section III—Gut

The gastrointestinal tract is home to the majority of human commensal bacteria, in what is called a “superorganism” [52]. This delicate balance is involved in numerous physiological functions that are essential for the body, and sometimes the onset of dysbiosis is enough to favour the development of fearsome clinical conditions, for example, *Clostridium difficile* infection [4]. The development of the microbiota starts from the earliest moments of life and is influenced by numerous environmental factors, among which cigarette smoke is also implicated [23,53]. For this reason, we asked how smoking affects the normal composition of the gut microbiota and, subsequently, whether these changes could be implicated in the onset of certain diseases.

The *Firmicutes*/*Bacteroidetes* ratio, uniformity, diversity, and abundance of the gut microbiota are important parameters for the composition of the gut flora [54].

The gastrointestinal tract is significantly affected by smoking; in fact, in most of the studies cited, there are differences in the composition of the microbiota between smokers and non-smokers [55,56,57,58,59]. This finding is also confirmed by a recent systematic review [60].

Several mechanisms have been proposed to explain this finding (Table 5). It has been shown that toxic substances from cigarette smoke, ingested in the gastrointestinal tract, can induce dysbiosis of the gastrointestinal microbiota through antimicrobial activity and regulation, altered mucosal immune responses, and increased mucosal permeability [55].

Despite some conflicting results, the main finding is a change in the proportion of the phyla *Bacteroidetes*, *Firmicutes,* and *Proteobacteria* in current smokers compared to never smokers, whereas no differences were found between former and never smokers [2,56,57,60,61].

As we have already mentioned, it is known that an increase in the *Firmicutes*/*Bacteroidetes* ratio correlates with a pro-inflammatory condition [51,55]. Based on these assumptions, we have found evidence in the literature of a smoke-mediated role on the microbiota, mainly in a few important topics which we will go on to discuss specifically, namely, IBD, gastrointestinal cancers, cardiovascular, and metabolic diseases.

Smoking is considered a notorious and ambiguous player in the pathogenesis of inflammatory bowel diseases (IBDs).

In Crohn’s disease (CD), there is increased permeability of the intestinal mucosal barrier, promoting chronic inflammation. Dysbiosis has been related both to the development and exacerbation of the disease. CD patients show a reduction of commensal phyla, such as *Bacteroidetes* and *Firmicutes*, and an increase of pathogens, such as *E. coli*, *Campylobacter* spp., and *Mycobacterium* spp. Smoke is known to worsen Crohn’s disease, and some authors have proposed microbiota-related mechanisms. For example, CD smokers have a reduction in specific genera—*Collinsella*, *Enterorhabdus*, and *Gordonibacter*—which produce molecules with anti-inflammatory properties. Moreover, the mucosa of CD smokers has lower levels of *Faecalibacterium prausnitzii*, a bacteria with immune-regulatory function, whose depletion has been correlated with a greater risk of recurrence following surgical resection in CD [8].

In ulcerative colitis (UC), colorectal dysbiosis is characterized by a low taxonomic diversity, a decrease in *Firmicutes*, and an increase in *Proteobacteria*. Moreover, the reduced abundance of *Bifidobacteria* is considered a microbial biomarker. Some authors have proposed that UC patients exhibit an aberrant mucosal immune response against their microbiota. While in CD smoking is a trigger factor, it is considered a protective factor in UC. In fact, smokers with UC are more likely to have milder disease, fewer hospitalizations, and reduced need for corticosteroid and immunosuppressants than non-smokers. Some authors have proposed that smoking could modify the microbiota composition, modulate mucus secretion, and impair mucosal healing. The results show how the studies have been contrasting, and other concomitant environmental and host factors (i.e., diet) have been involved [8].

The effect of smoking on the gut has been widely studied as a risk factor for cancer. Some authors have searched for a relationship between smoking and the microbiota in the pathogenesis of cancer, but the results have been contrasting [50,58].

With regard to the first tract of the digestive tract, there is scarce knowledge about the role of the microbiota, even if some authors have started to look for differences in the composition of the microbiota of smokers and non-smokers.

For example, Li et al. found that the saliva samples from non-smoking and non-drinking patients undergoing an upper gastrointestinal (UGI) endoscopic examination for UGI cancer screening had a greater abundance of *Neisseria*, *Prevotella*, *Porphyromonas*, and *Rothia*, and lower levels of *Streptococcus*, *Actinobacillus*, and *Haemophilus* than in the oesophagus. There were no significant differences in the quantity of most genera in the upper, middle, and lower oesophagus, and there were only slight differences in the microbiota between smoking and drinking individuals and non-smoking and non-drinking individuals [1]. These observations suggest that oral or oesophageal cancer caused by smoking and drinking may not be mediated by dysbiosis.

More is known about the interaction of smoke and the microbiota in the pathogenesis of colorectal cancer (CRC).

Starting from animal models, authors have reported that mice exposed to smoke have shown gut microbiota dysbiosis with a higher incidence of colorectal cancer. This was explained with increased pro-tumoral metabolites and impaired gut barrier function, which could activate oncogenic MAPK/ERK signalling in colonic epithelium [62].

It is known that in humans, CRC patients show enrichment in the abundance of *Streptococcus gallolyticus*, *Fusobacterium*, *B. fragilis*, and *Escherichia–Shigella*, while genera such as *Bacteroides*, *Roseburia*, and *Pseudomonas* were depleted. Smoking is a well-known factor involved in the initiation of CRC. Even if the mechanisms underlying the noxious effect of smoke in CRC need to be clarified, some authors have proposed a role of the ingestion of bacteria present in cigarettes [8].

Other studies have investigated the role of smoke-related gut dysbiosis in the pathogenesis of cardiovascular diseases, with contrasting results. Hu et al. reported a depletion of species belonging to *Bifidobacteria* and *Akkermansia*, and an enrichment of *Enterococcus faecium* and *Haemophilus parainfluenzae* in current smokers with coronary artery disease (CAD), compared with former and never smokers [63]. This resulted in changes in the microbiota-derived atherosclerosis-related metabolites, which were reversible after smoking cessation.

Sublette et al. reported that smoke abstention causes an increase in the relative abundances of the *Bacteroidetes* and *Firmicutes* phyla, correlating with heart rate, systolic blood pressure, and C-reactive protein. Moreover, they observed a relative abundance of *Actinobacteria* and *Cyanobacteria* in smokers, the first correlating with pack-years, and the latter with carbon monoxide levels [59].

Regarding its impact on metabolism, smoking has been related to glucose and lipid disorders [64,65].

Studies on animal models have revealed a modification of bile acid metabolism that, after smoke exposure, may be mediated by microbiota modifications. The expression of cholesterol synthesis-related genes was upregulated with the accumulation of total cholesterol (TC) in the liver. Moreover, the ratio of cholic acid (CA) to chenodeoxycholic acid (CDCA) was significantly reduced in the liver of mice exposed to cigarette smoke [64]. Furthermore, smoking induced hyperglycemia and significant reductions in serum insulin and leptin levels [65].

Interestingly, changes in the gut microbiota composition caused by the intestinal influx of smoke-related metabolites have been related to smoking addiction and failure of attempts to quit smoking. For example, smoking cessation-induced weight gain (SCWG) is considered a major obstacle to smoking abstinence. In a mouse model, smoke exposure led to a decrease of weight-lowering metabolites, such as N-acetylglycine. Microbiome depletion induced by treatment with antibiotics prevents SCWG, whilst faecal microbiome transplantation from mice previously exposed to cigarette smoke into germ-free mice never exposed to smoke causes weight gain, suggesting a role for microbiota modulation in the management of smoke dependence [66].

It has been reported that smoking cessation may lead to notable modifications in the composition of the microbiota; even in this case, contrasting results have been reported. For example, Biedermann et al. found an increased microbial diversity, with a higher abundance of *Firmicutes* and *Actinobacteria*, and a lower proportion of *Bacteroidetes* and Proteobacteria, in the stool of patients who quit smoking 9 weeks before [67,68].

### 3.4. Section IV—Other Organs

Smoking has been identified in observational studies as a risk factor for bacterial vaginosis (BV), characterized by a decrease of *Lactobacillus* spp. abundance. This has been linked to the anti-estrogenic effect of toxicants of smoking, which are found in the vaginal secretions of smokers.

In a pilot study, Brotman et al. enrolled women, 20 smokers and 20 non-smokers, to analyse their vaginal microbiota, and found that the first group had a lower proportion of vaginal *Lactobacillus* spp. They also studied the composition of the microbiota after smoking cessation, but they concluded that further studies are required to include smoking cessation as a strategy for reducing recurrent BV [69].

The microbiota, especially of the gut, has also been implicated in immune-mediated and neurodegenerative diseases.

For example, Sheperjans et al. explored the role of the “gut–brain axis” in the pathogenesis of Parkinson’s disease. It is known that there is an intense crosstalk between the gut microbiota and the nervous system influencing the brain, mainly mediated by levels of neurotransmitter receptors and neurotrophic factors. In Parkinson’s disease, a reduction in the abundance of the *Prevotellaceae* family was described. Moreover, the abundance of *Enterobacteriaceae* bacteria was related to the severity of postural instability and gait difficulty. The relevance of these bacterial families seems to be based on an increased mucosal permeability, and a subsequent increased systemic endotoxin exposure in PD subjects. Smoke has been reported to have neuroprotective effects. In smokers, a higher abundance of faecal *Bacteroides*/*Prevotella* was observed, and this abundance decreases after smoking cessation [70]. Regarding immune-mediated diseases, it is interesting to note that smoking may influence the response to therapies. Zhang et al. found not only that both smoking and TNF-α-blocker have a significant impact on the composition, relative abundance, and diversity of the gut microbiota in patients with ankylosing spondylitis (AS), but also the relative abundance of the gut microbiota showed fluctuations during treatment. Moreover, AS non-smokers showed a greater improvement rate compared to smokers [71].

## 4. Analysis and Limitations of Results

Since the research of the microbiota has been spreading in the last years, and smoking is one of the most studied risk factors for human disease, the number of works published on these topics is impressive.

For this paper, the authors decided to choose only three keywords that must be present in the titles of the articles. This has allowed us to obtain a very select group of articles, in order to try to precisely ask our questions: what is the relationship between smoking and the microbiota, what effect does it have on health and disease, and could we modulate these effects in order to prevent and even treat diseases?

Being this selective is a guarantee of pertinence, and we are aware that some valuable papers might not have been included in our work.

Even after choosing a twenty-five year interval for these articles selected in the preliminary search, almost all of them were published in the last ten years.

In particular, around three quarters of the articles were published in the last five years, indicating that the topic is coming to the attention of the scientific community and requires up-to-date discussion.

Most of the articles treated in this review are observational studies, with a prevalence of prospective and cross-sectional studies, and few retrospective studies.

Having then reviewed the strengths of this study, we turn to a discussion of its limitations.

The most important limitation is that in many studies the samples were small, with some exceptions [15,21].

The studies conducted on animal models, mainly on mice and rats, are of particular interest, since intentional exposure to smoke in humans—in order to conduct clinical trials—would be unethical. In fact, in the only two randomized clinical trials included in this review, the interventional action is the cessation of smoking.

The characteristics of the study exposed above have allowed us to extensively face two major issues of extreme topicality with regard to global health.

## 5. Discussion

Cigarette smoke is a classic risk factor for many diseases [72]. Recently, the microbiota has been indicated as a new, major player in human health [4]. Its deregulation—dysbiosis—is considered a new risk factor for several illnesses [4]. Since it has been reported that dysbiosis is promoted by many environmental factors, smoking included [23], we have been wondering if, besides its direct noxious effects on human cells, smoking may exert indirect harm by enhancing dysbiosis.

The growing interest in the microbiota has recently led to speculation that the negative effects of cigarette smoke may also manifest through the modification of the microbiota in a pathogenetic fashion. In our review of the literature, we have identified many mechanisms that have been suggested to explain these modifications.

First of all, authors have initially reported that cigarettes contain a variety of bacteria, including soil microorganisms, commensals, and potential human pathogens such as *Acinetobacter*, *Bacillus*, *Burkholderia*, *Clostridium*, *Klebsiella*, and *Pseudomonas aeruginosa*. These observations have been considered responsible for different bacterial communities in smokers [8].

Recently, other studies have described that smoking promotes dysbiosis through mechanisms such as antibiotic effects, oxygen deprivation, and biofilm formation. At the immune system level, smoking causes an immunosuppression characterized by a decreased activity of natural killer cells, and an altered phagocytic function of macrophages and neutrophils. All of these changes enhance the proliferation of pathogens, with the subsequent depletion of commensal species [24,25,73].

Since smoke goes directly through the oral cavity, the upper aero-digestive tract, and the distal airways, it obviously alters the composition of the microbiota in such regions. It is less clear how it may induce microbiota modifications in distant organs that do not have direct contact with the smoke. There is evidence that the toxicants of cigarette smoke, swallowed into the gastrointestinal tract, can induce gastrointestinal microbiota dysbiosis via different mechanisms, such as antimicrobial activity and regulation, impaired mucosal immune responses, and increased permeability of the mucosa [55].

In light of these observations, it comes as no surprise that many studies focusing on the identification of harmful effects of the interaction of smoke and the microbiota have been conducted on the oral cavity. In fact, the oral cavity hosts plenty of bacteria, even if 96% of the taxa belongs to the six major phyla *Firmicutes*, *Bacteroidetes*, *Proteobacteria*, *Actinobacteria*, *Spirochaetes*, and *Fusobacteria* [10].

Most of the works on the oral cavity are dedicated to the periodontal disease that we can consider as a “model”. The majority of the studies on periodontitis, the microbiota, and smoke share a common structure, comparing healthy versus periodontitis patients with different smoking habits (never smokers, former smokers, and current smokers).

**Figure 1:**
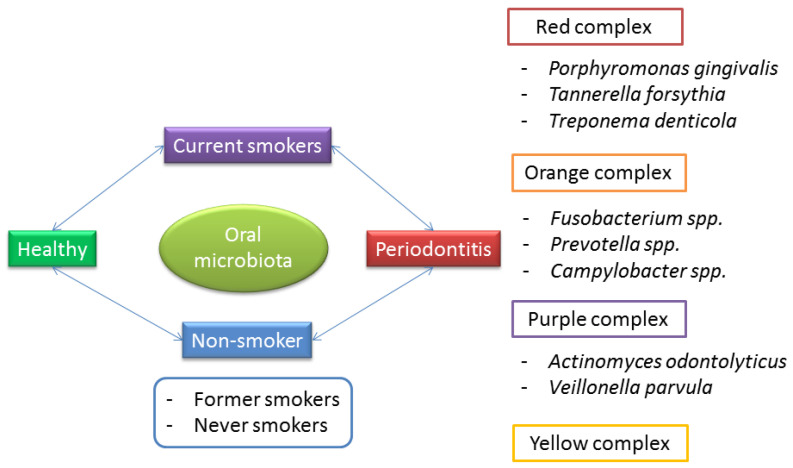
The interplay between smoke and the microbiota on the composition of the oral microbiota in the pathogenesis of periodontitis.

This structure allows many reflections. First of all, periodontitis is not an exclusive disease of smokers, and independently from the smoking habit, the role of dysbiosis in periodontitis is well established (Figure 1).

Classically, a triad of bacteria, called “the red complex”, is considered responsible for the pathogenesis of periodontitis: *Porphyromonas gingivalis*, *Tannerella forsythia*, and *Treponema denticola*. The “orange complex”, formed by *Fusobacterium*, *Prevotella*, and *Campylobacter* species, is also often associated with periodontitis [11,12]. Lastly, other bacteria have been reported to predispose one to the progression of periodontitis, and are grouped into the “purple” and “yellow” complexes.

It is interesting to note that these bacteria mostly belong to the phyla Bacteroidetes (Porphyromonas gingivalis, Tannerella forsythia, and Prevotella spp.), Spirochaetes (Treponema denticola), Fusobacteria (Fusobacterium), and Proteobacteria (Campylobacter).

In particular, *Bacteroidetes* is a phylum of Gram-negative bacteria. Although most *Bacteroides* spp. are symbiotic, and perform degradation of proteins or complex sugar polymers, some of them can be opportunistic pathogens. They are often counterposed to *Firmicutes*, a phylum of bacteria, most of which are Gram-positive commensals in the gut, and in the oral cavity too. This phylum includes two clinically relevant classes: the *Bacilli*, which are obligate or facultative aerobes, and the *Clostridia*, which are anaerobic. The class *Bacilli* includes the genus *Streptococcus*, whose members are the most abundant bacterial species in the mouth, and the family *Lactobacillaceae*, which are important probiotics [9,10].

The researchers who are confident with the microbiota know that one of the most studied markers of dysbiosis is the *Firmicutes*/*Bacteroidetes* ratio (F/B). In fact, it is known that a low *Firmicutes*/*Bacteroidetes* ratio is related to a pro-inflammatory condition, especially in the bowel [51,56].

Given these premises, we can assume that, regardless of smoking habit, periodontitis is promoted by dysbiosis, consisting of the depletion of beneficial bacteria, especially *Firmicutes*, and the proliferation of pathogens, especially *Bacteriodetes*, with a subsequent low F/B and pro-inflammatory status.

How does smoking affect this well-established model?

It has been reported that the microbiota of healthy smokers undergoes changes that resemble precursors of those of periodontitis, with higher levels of pathogens belonging to the purple, yellow, orange, and red complexes of periodontitis [11,12]. Moreover, smokers who still have periodontitis show an even less diversity of bacteria and pathogen richness, correlating with higher disease severity than non-smokers [12,16]. Finally, there is contrasting evidence regarding the effects of smoking cessation. Some authors have reported that smoking cessation is associated with restoration of the normal oral microbiota [21], while other researchers have demonstrated that the imbalance may persist, even many years after smoking cessation [29].

The information we obtained in this review of the recent literature suggests that, besides an independent role of smoke on oral cavity health, and an independent role of dysbiosis, there is a third, conjunct mechanism, that can be imagined as a *continuum*: starting from the exposure of a healthy microbiota to the deleterious effects of smoke, the arising of pathogens and depletion of beneficial commensals, the establishment of a pathogenic pro-inflammatory microbiota, and disease appearance and progression to more severe forms in smokers than in non-smokers. This model seems to be valid, not only for periodontitis, but also for other oral illnesses, such as caries [25] and aphthous stomatitis [19].

It is our opinion that the differences existing among the studies rely on the heterogeneity of the numerosity of the study population, inclusion and exclusion criteria, the site and method of specimen collection, and processing techniques.

In particular, it is difficult to compare studies of different anatomical sites, since it is possible that they offer different living conditions to bacteria, such as different oxygen tension, adhesion surface, and immune system interaction.

Moreover, the definition of “smoking habit” is heterogeneous too, and studies take into account different confounding factors, such as demographics, existence and duration of smoking habit, severity of periodontal disease, and coexisting risk factors, such as diet habits, obesity, oral hygiene, chronic illnesses and the related chronic therapy, included antibiotics.

It is our opinion that it is undeniable that a role for the interaction of smoke and the microbiota in the oral cavity pathology exists. Wider and more homogeneous studies are required to perform better comparisons, and to better understand how this interaction can be useful in the future. In particular, this knowledge may be of pivotal importance to exploit all the potential tools we yet have at our disposal to prevent and treat these diseases, such as smoking cessation, prebiotics, and probiotics.

With regard to the airways, our first consideration is that those diseases in which a role for smoke–microbiota interaction has been reported are not exclusive to smokers. That is why studies have been structured in the same fashion of the oral cavity, comparing healthy and ill subjects with different smoking habits.

For example, COPD and lung cancer can be promoted by exposure to air pollutants, even some related to work activities; asthma also affects individuals who were never exposed to passive smoke; and cystic fibrosis depends mostly on genetic factors [74,75,76]. It is also established that smoking—both active and passive—is a factor that promotes the onset, progression, exacerbation, and severity of these chronic diseases [8]. That said, the microbiota of such patients shows differences, not only with respect to the healthy population, but also with respect to the smoking habit [44]. It has been reported that healthy smokers show a reduction of commensals, and an increase of pathogens, with a microbiota profile that resembles the one observed in chronic respiratory diseases [44]. So, even in the case of airways, it is possible to imagine the *continuum* of alterations that has been previously assumed for the oral cavity.

Again, this being a logical hypothesis, and supported by some valuable works, we believe that the evidence is too weak, scarce, and contradictory to support this conjecture. This weakness relies on some intrinsic limitations of the systems studied, but also on the heterogeneity of the study designs.

The respiratory system includes organs that are very different regarding position, communication with the external environment, microanatomical structure, and physiological function. For these reasons, there is still not any agreement on the techniques used to study the airway microbiota, especially the one of the distal respiratory tract and lungs. That is why, even if there is evidence suggesting that most of the species colonizing the airways originate from the aspiration of the oral ones, the presence of peculiar species suggests a role for contamination during the collection of the specimen, or from other regions, such as the throat or the gut [32].

Moreover, as previously reported for studies of the oral cavity, there is heterogeneity in the numerosity of the population, inclusion/exclusion criteria, definition of the existence and duration of the smoke exposure, concomitant evaluation of the oral and general health status, correction for confounding factors, and sampling and sequencing techniques.

Additionally, we noticed that only a few studies take into account the season, the geographic place, and the socio-economic status of patients [33]. We believe that these factors deeply affect the quality of the breathed air—and so of the type and concentration of microorganisms and pollutants—that may contribute in a relevant way to the alteration of the airway microbiota.

That said, with particular reference to COPD, we want to highlight that most of the studies analyzed the composition of the microbiota in nasal samples. We suggest that this sampling may not reflect the actual composition of the distal airways and alveoli; that is, the anatomical districts where we observe the pathognomonic histopathological alterations of the disease and its exacerbations. For these reasons, many of the considered studies have been conducted on animal models [47,48].

Even with all these study design limitations, and in the presence of scarce data, we can recognize some common patterns. The upper airway microbiota in smokers shows an increased relative abundance of *Firmicutes* and *Actinobacteria*, and a decrease in *Proteobacteria* abundance [33,44]. The lungs of smokers have a higher abundance of *Proteobacteria*.

All of these critics suggest that more studies are needed to better understand the role of smoke–microbiota interplay in the respiratory pathology, and to use, in the future, not only smoking cessation but also antibiotics, prebiotics, probiotics, and vaccines (such as the one against *Haemophilus influenzae*) to open new preventive and therapeutic possibilities.

Smoking is implicated in the pathogenesis of several gastrointestinal diseases, such as IBD, neoplasms, and metabolic disorders. Several studies agree that smoking alters the composition of the gut microbiota [55,56,57,58,59,60]. For this reason, we wondered if it was smoke-induced dysbiosis that promoted the development of these diseases.

With regard to intestinal bowel diseases, we can state that smoking plays a key dual role in their development, as smoking appears to play a protective role in ulcerative colitis (CU), while it is a risk factor for Crohn’s disease (CD), though the mechanisms underlying this relationship appear to be intricate [77,78]. The lack of solid results relies on the fact that IBDs are extremely complex diseases with a multifactorial pathogenesis, so that it is extremely difficult to put confounding factors on the back burner.

There is no doubt that smoking is one of the most important risk factors for the occurrence of gastrointestinal malignancies [79], so, we wondered about the potential role of the microbiota–smoking interplay. With regard to tumours affecting the first tract of the gastrointestinal system, such as those of the oral cavity or oesophageal tract, there seems to be no correlation with dysbiosis [58]. Probably, in this case it is the harmful products related to smoking that favour the onset of neoplasms [79]. On the contrary, there is evidence supporting the involvement of dysbiosis with the onset of colorectal cancer. Several underlying mechanisms have been proposed, e.g., smoking seems to deplete the amount of *Bifidobacterium*, resulting in a reduction of butyrate, an anti-inflammatory and anti-cancer molecule. According to some authors, smoking-induced dysbiosis may also alter the composition of mucus layers, resulting in inflammation, a known risk factor for colorectal cancer [8]. In this case, it is therefore hypothesized that it is the negative effect of smoking, mediated through dysbiosis, that plays an important role in carcinogenesis, although further studies are needed.

The gut microbiota also appears to play an important role in the development of cardiovascular diseases, such as atherosclerosis [80]. Again, the pathogenesis could be mediated by smoking-induced dysbiosis.

In light of these observations, some authors have proposed a role of antibiotics, prebiotics, probiotics, and faecal transplantation in the restoration of gut eubiosis in smokers, even though most of the studies have been conducted in animal models [66,81].

We would like to point out that the treatment in four separate paragraphs was chosen solely to facilitate the discussion of this topic. However, in addition to an exquisitely local role in regulating homeostasis, it is important that the microbiota is a network that also includes ‘paracrine’ communication between the resident bacterial populations in the body. The different bacterial populations, in fact, interact with epithelial cells and the immune system, and all these cells release substances—e.g., SCFA, interleukins—that when released into the bloodstream, reach distant organs. It is precisely for this reason that the ‘gut–lung axis’, the ‘gut–brain axis’, and others have been described [82,83].

It is safe to assume that, even if studies on this are still few and preliminary, intestinal dysbiosis may play a role in respiratory pathology and vice versa [84], just as this could potentially be true for other organs.

Confirming these preliminary data with future studies could be vital for the rational use of prebiotics and probiotics.

**Figure 2:**
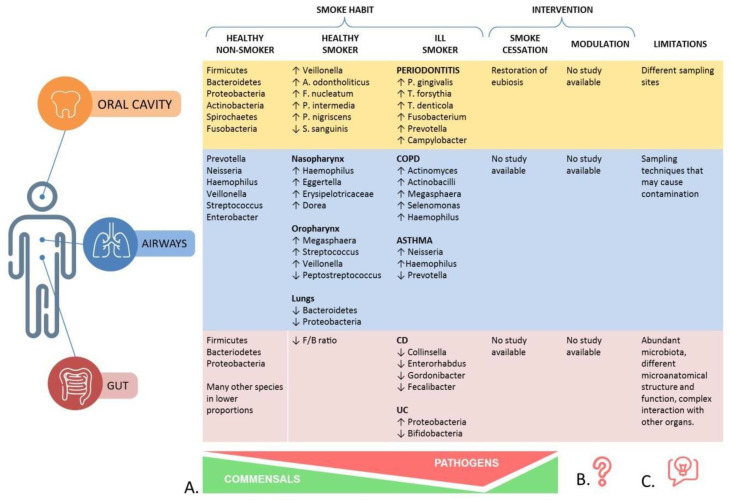
Main findings of this review. (**A**) The model of smoke-related dysbiosis in different body regions, characterized by a progressive depletion of beneficial commensals and an increase of pathogens. (**B**) Effects on smoke-related dysbiosis after different interventions, such as smoking cessation and modulation of the microbiota composition (prebiotics, probiotics, fecal transplantation). (**C**) Main limitations found in the design of studies available on the topic.

To our knowledge, this is the first article that, based on the results found for periodontitis, assumes the existence of a model of smoke-driven microbiota alterations, and detects evidence supporting the application of this model to other organs and related pathologies. In this model, the microbiota of the healthy smoker is a precursor to the one observed in pathologic conditions. The persistence of smoke-related noxious effects in ill patients further affects the microbiota composition, with a depletion of commensals and an increase of pathogens, which correlates with the severity of the illness and the recurrence of exacerbations (Figure 2A). The importance of describing a common pathway of smoke-related dysbiosis relies not only on the considerable help it gives in understanding the results of existing studies, but also for the design of future studies.

The other important contribution of our review is to have highlighted the heterogeneity of study designs, which affects the comparability of the results. These observations should certainly be taken into account when studying the same topic in the future (Figure 2C).

Finally, one of the first intents of this review was to explore the evidence supporting the use of interventions such as smoking cessation, and modulation of the microbiota with prebiotics, probiotics, FMT, and other strategies in smoke-dysbiosis related diseases. Although there are grounds for some applications [84,85], in terms of both prevention and treatment, to work in theory, mainly when considering the potential restoration of eubiosis after smoking cessation, there is currently no solid evidence. The fact that we have not been able to find studies on the topic could be seen as a weak point of this review, yet we believe that this is one of the most interesting findings. In fact, we have brought to light a great lack of studies on the topic, that, given the great number of studies on smoking and the microbiota, we did not expect to find. We can, therefore, say that this certainly remains an important area to be explored, and our study shows that it is still at the stage of hypothesis at the moment (Figure 2B).

## 6. Conclusions

Cigarette smoking may affect organs in many ways. In addition to direct damage caused by toxic combustion products, indirect damage has been described. Among the indirect mechanisms, dysbiosis seems to be involved.

The microbiota has been identified as a major player in many diseases, by involving not only the gut [4], but also distant organs [5,6]. The microbiota participates in the human body homeostasis, and its composition can be perturbed by many life habits, including nutrition, antibiotics, and smoking. Interestingly, studies suggest that smoking may affect bacterial microbiota cells with the same negative effects that it exerts on human cells. More surprisingly, this deregulation of the microbiota affects not only those organs that are in direct contact with the smoke, such as the oral cavity, contiguous cavities, and the upper and lower airways, but in fact, distant organs such as the intestine, heart, vessels, and genitourinary tract may also be affected. These observations yield a deeper insight into the mechanisms involved in the pathogenesis of smoke-related disease, suggesting a role of dysbiosis, with a recurrent pattern of a depletion of beneficial bacterial species and a proliferation of pathogens. Even if we are far from using probiotics as a systematic and rational therapeutic approach [6], we may speculate that the modulation of the microbiota may help in preventing and treating some of these illnesses in the future.

We believe that our review is relevant because it demonstrates that even if the interaction between smoking and the microbiota has been widely studied in the last twenty years, we are far from understanding it unequivocally.

In fact, many of the studies analyzed in our paper have reported conflicting results. This demonstrates that further studies may be useful to better define the cross-interaction between smoking and dysbiosis, and its possible applications in clinical practice.

## Figures and Tables

**Table 1:** List of inclusion and exclusion criteria.

Inclusion Criteria	Exclusion Criteria
Type of article: in order of importance, we have considered clinical trials, observational studies, systematic reviews, narrative reviews.	Topic: articles regarding only the topic “microbiota” or only the topic “smoking”; articles treating other topics.
Language: only articles written in English.	
Year of publication: articles written in the last 25 years.	

**Table 2:** List of the articles treated in the discussion divided in the four groups, and corresponding references.

Group	Total Articles	References
Oral cavity	24	[8,9,10,11,12,13,14,15,16,17,18,19,20,21,22,23,24,25,26,27,28,29,30,31]
Airways	21	[32,33,34,35,36,37,38,39,40,41,42,43,44,45,46,47,48,49,50,51,52]
Gut	16	[53,54,55,56,57,58,59,60,61,62,63,64,65,66,67,68]
Other Organs	3	[69,70,71]
Total	64	

**Table 3:** Main studies conducted on the relationship between smoking and the oral cavity microbiota composition.

Features of the Study	Conclusions	References
82 patients, 22 non-smoking healthy controls, 28 non-smoking periodontal patients, and 32 smoking periodontal patients	Patients with periodontal disease have greater bacterial diversity, and non-smokers have a more diverse microbial community	Camelo-Castillo, A.J. et al., 2015 [12]
32 smoking and 32 non-smoking	Smoking affects the composition of the subgingival microbiota	Bašić, K. et al., 2021 [14]
316 healthy subjects	Smoking alters the composition of the microbiota of the oral cavity	Jia, Y. J. et al., 2021 [15]
558 participants divided into non-smokers, cigarette smokers, opium smokers, or both	Cigarette and opium smoking is associated with lower alpha diversity and a different composition of the oral microbiota	Wu, Z. et al., 2021 [20]
Saliva samples donated by 41 people	Evidence of a different composition of the oral microbiota between healthy patients and those with periodontal disease, which in turn diversified, whether smokers or non-smokers	Grant, M. et al., 2019 [27]
30 individuals, 10 tobacco smokers, 10 electronic cigarette users, and 10 controls.	Diverse composition in tobacco smokers, no significant differences between alpha or beta diversity, or in relative taxonomic abundances between e-cigarette users and controls	Stewart, C. J. et al., 2018 [30]

**Table 4:** Main studies on the link between smoking and the airway microbiota composition.

Features of the Study	Conclusions	References
29 smokers and 33 healthy adults	In smokers, the airway microbiota was significantly more diverse	Charlson, E. S. et al., 2010 [38]
11 patients with COPD associated with tobacco smoke, 10 with COPD associated with biomass smoke, 10 healthy individuals	Evidence of microbial community dysbiosis among different groups	Agarwal, D. M. et al., 2021 [44]
74 patients admitted after severe blunt trauma	Smokers had a significant increase in potential pathogens	Panzer, A. R. et al., 2018 [49]

**Table 5:** Main studies conducted on the relationship between smoking and the gut microbiota composition.

Features of the Study	Conclusions	References
33 smokers and 121 non-smokers	Smoking significantly affected the gut microbiota	Yan, S. et al., 2021 [55]
758 men, divided into never smokers, former smokers, and current smokers	Different composition of gut microbiota between smokers and non-smokers; no difference between smokers and ex-smokers	Lee, S. H. et al., 2018 [56]
102 patients undergoing endoscopy	Both smokers and ex-smokers show reduced bacterial diversity in the small intestinal mucosa of the upper intestine compared with those who have never smoked	Shanahan, E. R. et al., 2018 [57]
76 healthy participants	Finding of taxonomic and microbial diversity in the oral and esophageal cavity among non-drinkers and non-smokers compared with drinkers and smokers	Li, Z. et al., 2021 [58]
36 patients who were trying to quit smoking	Minor changes were found once smoking was stopped	Sublette, M. G. et al., 2020 [59]
Systematic Review	In most works, a decrease in both bacterial species and variability indices was found in fecal samples from smokers	Antinozzi, M. et al., 2022 [60]

## Data Availability

The articles cited in this paper are available on PubMed^®^, UptoDate^®^, and Cochrane^®^.
